# Enhancing Visuospatial Working Memory and Motor Skills Through School-Based Coordination Training

**DOI:** 10.3390/sports13110396

**Published:** 2025-11-06

**Authors:** Pasqualina Forte, Elisa Pugliese, Giovanna Aquino, Carmela Matrisciano, Fabio Carlevaro, Francesca Magno, Daniele Magistro, Cristiana D’Anna

**Affiliations:** 1 Department of Education and Sports Sciences, Pegaso University, 80143 Napoli, Italy; pasqualina.forte@unicam.it (P.F.); elisa.pugliese@unicam.it (E.P.); carmela.matrisciano@univr.it (C.M.); 2 Center for Neuroscience, University of Camerino, 62032 Camerino, Italy; 3 Department of Medicine and Health Sciences, University of Molise, 86100 Campobasso, Italy; giovanna.aquino@unimol.it; 4 Department of Neuroscience, Biomedicine and Movement, University of Verona, 37129 Verona, Italy; 5 Asti Higher Studies University Pole, Uni-Astiss, 14100 Asti, Italy; fabio.carlevaro@unito.it; 6 Department of Life Sciences and Systems Biology, University of Turin, 10124 Turin, Italy; francesca.magno@unito.it; 7 School of Health Science, Faculty of Environmental & Life Sciences, University of Southampton, Southampton SO17 1BJ, UK; d.magistro@soton.ac.uk

**Keywords:** visuospatial working memory, executive functions, motor-cognitive integration, motor coordination, school intervention, physical activity, physical education curriculum

## Abstract

The school-age period is a crucial time for the integrated development of cognitive and motor functions. Literature highlights that physical activity enhances executive functions, including visuospatial working memory (VSWM). In light of this evidence, this study investigated the effects of a school-based coordinative motor intervention on VSWM and gross motor skills in primary school children. An experimental research trial was conducted involving 184 children aged 9–10 years (mean age = 9.5 years, SD = 0.50 years), with 51.1% girls, divided into an experimental group (EG; *n* = 110), and a control group (CG; *n* = 74). Randomisation was performed at the class level via sealed envelope extraction by an independent researcher, ensuring allocation concealment. Outcome assessors were blinded to group assignment. VMWM was assessed using BVS-Corsi-2, and gross motor skills were evaluated via the TGMD-3. The EG showed significant improvements in VSWM (Corsi Forward: *p* < 0.001, d = 1.12; Corsi Backward: *p* < 0.001, d = 1.40) and gross motor skills, including Total Gross Motor: *p* < 0.001, d = 1.58, as well as in locomotion (*p* < 0.001, d = 2.11) and ball skills score (*p* < 0.001, d = 1.34). These findings strongly endorse incorporating cognitively demanding physical activities into standard school programmes to support children’s overall development and demonstrate the practicality of implementing such programmes within existing educational settings.

## 1. Introduction

School age represents a critical period for the integration of cognitive and motor functions [1]. During this developmental stage, both neurological maturation and environmental factors play a pivotal role in shaping cognitive abilities and motor competencies [2]. Notably, cognitive and motor skills are underpinned by shared mechanisms, such as planning and sequencing, and follow parallel developmental trajectories [3]. These overlapping developmental pathways are particularly evident in tasks requiring both cognitive control and motor coordination, such as those involving balance, rhythm, or spatial navigation—highlighting the close interplay between executive functioning and movement. However, despite this strong developmental interconnection, children are increasingly falling short of recommended physical activity levels. According to the Global Matrix 4.0 report, only 25% of children and adolescents worldwide meet the minimum guidelines for daily physical activity [4,5,6,7]. This decline—driven by sedentary lifestyles, screen-based behaviours, and limited opportunities for active play—raises concern not only for physical health but also for cognitive development. Regular physical activity plays a critical role in supporting both domains. Importantly, increasing the time allocated to physical activity within the school setting not only fails to hinder academic achievement but has been shown to enhance executive functions and learning outcomes. Children who engage in regular physical activity consistently exhibit superior physical and cognitive health compared to their less active peers [8].

Cognitive functions refer to the set of mental processes that enable individuals to acquire, process, store, retrieve, and utilise information. These processes underpin adaptive behaviour, learning, and interaction with the environment [9]. During childhood, the brain exhibits high levels of plasticity and responsiveness to environmental stimuli. In this context, motor experiences, particularly those involving coordination, have been shown to stimulate neural circuits that support both motor control and executive functioning. Emerging evidence suggests that coordinative motor activities can promote the maturation of brain regions associated with cognitive processing, particularly the prefrontal cortex and cerebellum, which are involved in executive function, working memory, and attention regulation [10,11,12]. Executive functions (EFs) are higher-order cognitive processes that govern the voluntary control of behaviour, thoughts, and emotions, primarily mediated by the prefrontal cortex. They encompass three core skills: inhibitory control, working memory, and cognitive flexibility [10,13,14]. These skills are essential for regulating thoughts, emotions, and behaviours to achieve specific goals, and they are particularly critical in complex situations that demand reasoning, planning, and decision-making capabilities [15].

Visuospatial working memory (VSWM), a core component of EFs, is particularly relevant when considering the cognitive demands of movement [16]. It refers to the ability to temporarily store, update, and manipulate visual and spatial information, such as remembering object locations or mentally navigating through space [16,17,18]. This function is fundamental not only to academic learning, such as geometry, handwriting, or reading maps, but also to planning, executing, and adapting goal-directed motor actions in dynamic environments. Among EFs, working memory plays a central integrative role, acting as a foundational mechanism that supports the effective operation of inhibitory control and cognitive flexibility. It enables children to maintain spatial goals, integrate visual feedback, and adapt movements in response to changing task demands, thereby facilitating more coordinated and purposeful motor behaviour. This function involves not only retaining visuospatial representations but also engaging in their active transformation to guide future action. It operates in close interaction with inhibitory control (to resist distractions) and cognitive flexibility (to adapt strategies), highlighting the interdependent and dynamic nature of EFs [19,20]. Recent evidence suggests that VSWM, in particular, is closely tied to the performance of complex motor tasks that require timing, rhythm, balance, and spatial accuracy. These coordinative demands engage shared neural resources in the prefrontal cortex, cerebellum, and parietal areas, regions implicated in both movement planning and spatial cognition [12,21,22,23].

Previous research has demonstrated a strong association between working memory skills and school readiness, as well as academic success in core subjects such as mathematics and reading [24]. Even at the preschool level, children with strong working memory abilities tend to perform better in acquiring foundational numerical and linguistic competencies [25,26]. Growing interest in enhancing working memory has led to the development of a wide range of educational programmes and psychopedagogical interventions. Diamond [15] highlights the importance of sustained practice and increasing task complexity to drive long-term gains in executive functioning. Nonetheless, existing evidence points to a key limitation: such cognitive training often produces effects that are domain-specific and may not generalise across broader executive function domains [27].

Working memory also plays a central role in educational policy. As emphasised by Moffitt et al. [28], children who demonstrate stronger self-regulation and working memory skills during early childhood tend to experience better long-term outcomes across multiple domains, including health, employment, interpersonal relationships, and reduced engagement in risky behaviours. This highlights the strategic importance of targeting working memory early in development, particularly through evidence-based, school-integrated approaches. Diamond [29] reinforces the importance of integrating evidence-based educational practices that support the development of working memory from early childhood. This includes implementing appropriate pedagogical approaches, providing consistent emotional support, and creating stimulating learning environments that nurture executive function development.

Physical activity is likewise a fundamental component of a child’s holistic development, exerting a significant influence on both physical and psychosocial growth [30]. Scientific evidence indicates that engaging in physical activity during the early years of life serves as a protective factor for long-term health, with benefits that extend well into adulthood [31]. Moreover, regular engagement in structured physical exercise has been shown to strengthen both fundamental motor skills and higher-order cognitive processes, including working memory [32,33].

Strong motor competence, particularly in coordination, is foundational not only for physical proficiency but also for socio-emotional development and participation in lifelong physical activity. Conversely, poor motor skills are associated with reduced physical activity participation and lower confidence. The acquisition of such skills is not automatic; rather, it depends on intentional, structured practice within stimulating learning environments. Hence, developmentally appropriate school settings that offer rich sensorimotor and social experiences are essential in fostering optimal physical and cognitive outcomes [34].

While many school-based interventions emphasise aerobic activity [35,36,37], coordination-based training may provide distinct cognitive advantages [38,39] due to its greater demand for motor planning, rhythm, and inter-limb control. These tasks inherently engage executive functions and require higher attentional resources than simple aerobic routines.

Childhood is thus a time of rapid and interconnected development across motor, physical, and cognitive domains [1,40]. Within the context of educational policy and curriculum design, it is crucial to acknowledge the formative and interdisciplinary value of physical education as a core subject that supports the holistic development of children—encompassing physical, cognitive, emotional, and social domains [30,41,42]. Consequently, it is essential to provide well-structured physical education programmes that not only support physical health but also foster emotional and social well-being, thereby encouraging children’s sustained engagement in physical activity [43].

Beyond the well-established benefits of aerobic exercise [44], recent research highlights the unique cognitive benefits of coordination-based physical activity [45,46,47]. Coordination exercises involve complex motor tasks that require the simultaneous use of multiple body segments and degrees of freedom in pursuit of specific goals [48]. These activities stimulate neuromotor adaptation at both peripheral (e.g., neuromuscular control) and central (e.g., activation of specific brain networks) levels, potentially enhancing cognitive performance [49].

Studies in children have shown that coordination-based physical activity is positively correlated with cognitive performance, likely due to increased activation of the cerebellum and neurobehavioural systems involved in executing complex motor tasks [50,51]. Despite these promising results, there is still limited evidence on the long-term effects of such interventions, particularly in relation to dose, intensity, and duration.

In light of the growing evidence linking coordination-based physical activity with cognitive enhancement, the present study aims to evaluate the effectiveness of a structured motor intervention in improving both visuo-spatial working memory and gross motor skills among primary school children. While inhibition and cognitive flexibility are equally important components of executive function, the current study focused solely on VSWM due to feasibility constraints, testing time, and appropriateness for school-based group administration. Specifically, the study examines whether participation in a school-based coordination training programme leads to significantly greater improvements in these domains compared to a standard physical education control condition. It is hypothesised that children in the experimental group will demonstrate higher performance in VSWM tasks and exhibit greater gains in gross motor competence compared to their peers in the control group.

## 2. Materials and Methods

### 2.1. Design

An experimental research design was employed in this study, involving one experimental group and one control group to evaluate the effects of a coordinative exercise intervention. The intervention was conducted in a primary school located in Benevento, Southern Italy, and included pupils from Year 4 and Year 5 classes. The participating classes were randomly assigned to either the experimental or control group. Randomisation was conducted at the class level using sealed envelope extraction by an independent researcher not involved in testing or intervention delivery.

Data collection commenced following formal authorisation from the host school, which approved the implementation of the research project. Prior to the intervention, a preliminary meeting was held with the teaching staff to outline the study’s objectives, methodology, and procedures, thereby ensuring a shared understanding and securing the full cooperation of the school personnel.

This study received approval from the Ethics Committee of the Pegaso Digital University (001221) and followed the principles outlined in the Declaration of Helsinki.

Written informed consent was obtained from the parents of all participants, and assent for participation was provided by the children after an age-appropriate explanation of the study.

### 2.2. Participants

This study involves 184 children aged 9–10 years old (mean age = 9.5 years, SD = 0.50 years), 51.1% girls and 48.9% boys, divided into an experimental group (EG; *n* = 110) and a control group (CG; *n* = 74). Descriptive characteristics of the participants are presented in Table 1.

Based on prior literature, a power analysis was conducted to determine the required sample size. We estimated a medium effect size (f = 0.25), with α = 0.05 and power = 0.95, which indicated that at least 158 participants would be needed. Our final sample of 184 children exceeded this requirement, ensuring sufficient statistical power for the analyses.

**Figure 1:**
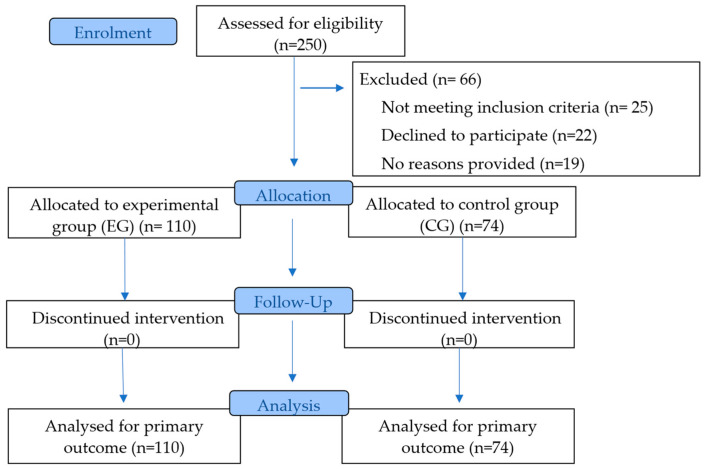
CONSORT 2025 Flow Diagram.

Figure 1 presents the CONSORT 2025 flow diagram [52], detailing the phases of the two-arm randomized trial, including participant enrolment, intervention allocation, follow-up, and data analysis. Of the 250 subjects assessed for eligibility, 66 were excluded due to failure to meet inclusion criteria, refusal to participate, or absence of a specified reason. Among the remaining participants, 110 were allocated to the experimental group and 74 to the control group. No participants discontinued the intervention during the follow-up period, and all subjects assigned to both groups were included in the analysis of the primary outcome.

### 2.3. Tools

#### 2.3.1. BVS-Corsi-2 Battery for the Assessment of Visuospatial Working Memory (Ages 8–12)

VSWM was assessed through the Corsi Block-Tapping Task, specifically utilizing the version integrated within the BVS-Corsi 2 [53]. This task is a recognized instrument for evaluating short-term memory, visuo-spatial working memory, and attentional control [54]. Recent investigations by Mammarella et al. (2023) [53] have further elucidated the utility of this assessment in understanding its relationship with working memory, executive functions, and spatial processing capabilities across diverse populations, including pediatric, geriatric, and cognitively impaired cohorts. The equipment comprises a board with nine wooden blocks, numbered 1 to 9, arranged in a non-linear configuration in front of the participant. During each trial, the examiner taps a predetermined sequence of blocks, beginning with two-block sequences and progressively increasing in length, following a standardized protocol. After each demonstration, the participant is instructed to reproduce the sequence exactly as observed. For each sequence length, three trials are presented; progression to the next level occurs if at least two of the three sequences are correctly reproduced. The assessment includes two tasks: the Corsi Forward task and the Corsi Backward task. In the Forward task, participants reproduce the sequence in the same order as demonstrated. In the Backward task, they must recall the sequence in reverse order, thereby imposing greater cognitive demands and engaging additional executive functions such as inhibition and cognitive flexibility. Test administration for each condition is discontinued after three consecutive errors at a given sequence length. The total duration of the assessment, including instructions and practice trials, is approximately 15 min per participant.

#### 2.3.2. Test of Gross Motor Development—3rd Edition (TGMD-3)

Gross motor skills are goal-directed movement patterns involving large whole-body movements [55], locomotion, and whole-body stretches [56]. Fundamental motor skills represent a subset of gross motor skills and are the foundation of physical education curricula worldwide [57]. These skills can be broadly categorised into two key areas: locomotion, which includes skills such as running, jumping, and hopping, and object control, which encompasses actions like throwing, catching, and kicking. This variation stems from a complex interplay of biological factors and environmental influences [1,58,59,60,61,62,63,64,65,66,67]. To systematically evaluate these essential skills, we employed the Italian version of the Test of Gross Motor Development-Third Edition (TGMD*-3*) [68,69,70,71]. This standardized, norm-referenced assessment tool is specifically designed for children between 3 and 11 years of age. The TGMD-3 provides a comprehensive evaluation through two distinct but complementary subtests. The first sub-test focuses on locomotor skills, evaluating six fundamental movement patterns: running, galloping, hopping, skipping, horizontal jumping, and sliding. The second sub-test examines ball skills or object control, assessing seven key manipulative abilities: two-hand striking, one-hand striking, dribbling, catching, kicking, overhand throwing, and underhand throwing. Each skill is evaluated using a detailed evaluation, where a score of 1 indicates correct execution and 0 indicates the criterion was not met. To ensure reliability and consistency, children complete two trials for each task. The scores from both sub-tests are then combined to generate the Total Gross Motor, which provides a comprehensive, quantitative measure of a child’s overall motor proficiency. In practical terms, the assessment is administered individually to allow for precise observation of movement quality and technique. While the standard administration time is approximately 20 min per child, this may vary depending on factors such as the child’s age and the examiner’s level of experience.

#### 2.3.3. Previous Day Physical Activity Recall (PDPAR)

Data on extracurricular physical activity were collected using the Previous Day Physical Activity Recall (PDPAR) questionnaire [72], a validated, self-administered tool for assessing physical activity (PA) in children and adolescents. It requires participants to recall activities performed on the previous day during after-school hours (3:00 p.m. to 11:30 p.m.), dividing time into seventeen 30 min intervals. For each time block, participants select their main activity from a predefined list of coded options and indicate the perceived intensity of that activity. To aid in this process, the PDPAR includes pictorial representations of four relative intensity levels: light, moderate, hard, and very hard.

#### 2.3.4. Anthropometrics

Body weight and height were measured at baseline to calculate participants’ body mass index (BMI). Weight was recorded to the nearest 0.5 kg and height to the nearest 0.1 cm using a digital scale and a stadiometer, respectively. BMI was calculated as weight in kilograms divided by the square of height in meters (kg/m^2^).

### 2.4. Training Protocol

A 16-week intervention programme based on coordination exercises was implemented specifically for school-aged children. The sessions were held twice weekly and were integrated into regular physical education (PE) classes.

All intervention sessions were supervised by trained PE teachers. A fidelity checklist was applied to 20% of the sessions, showing 95% adherence to the planned protocol. Inter-rater reliability for TGMD-3 scoring was assessed on 15% of the sample (ICC = 0.92), indicating excellent reliability. For the calculation of inter-rater reliability (ICC = 0.92) of 15% on the sample, two independent trained assessors observed and scored the TGMD-3 performances, indicating excellent reliability.

At baseline, the Previous Day Physical Activity Recall (PDPAR) was administered to assess physical activity levels, and anthropometric measurements, were collected for the calculation of Body Mass Index.

Before and after the intervention, all participating children underwent standardised motor assessments to measure gross motor skills at baseline and post-intervention. In parallel, cognitive assessments were conducted to evaluate visuospatial and working memory skills. The baseline and post-intervention assessments were administered by trained evaluators who were not involved in the delivery of the intervention, in order to ensure assessment independence and minimize potential bias.

Each individual assessment session lasted approximately 45 min per participant. Each training session, lasting 60 min in total, followed a structured format. The introductory phase (5–10 min) involved a warm-up comprising low- to moderate-intensity aerobic activities, with an emphasis on coordination involving both upper and lower limbs. This was followed by a central phase (40 min) dedicated to coordination-focused exercises. The session concluded with a short final phase (approximately 5 min), during which participants reviewed and reflected on the activities, often through playful group games or “circle time”, aimed at consolidating learning and encouraging metacognitive engagement. The experimental group followed a structured coordination training protocol characterised by progressive increases in intensity and complexity. This approach is grounded in the principle of systematic variation in motor practice, whereby task demands are gradually intensified to stimulate neuromotor development [73,74,75].

The protocol, tailored specifically for this age group, encompassed a diverse array of activities including rule-based and strategic games, rhythmic exercises designed to enhance temporal perception, and movement sequences performed with or without music and equipment. Specifically, the training included: dexterity circuits, rope exercises, throwing and catching drills, static and dynamic balance activities, jumping and directional change exercises, rhythm and timing tasks, hand-eye and foot-eye coordination drills, motor response activities, and exercises targeting motor differentiation. Particular emphasis was placed on space-time orientation in relation to the surrounding environment. The intervention was delivered by a Sports Science graduate under the supervision of the class PE teacher, in accordance with the objectives outlined in the Italian National Curriculum for Primary Education, which emphasises the development of “*self-awareness through body perception and mastery of motor and postural patterns in continuous adaptation to spatial and temporal variables”* [76]. The intervention protocol programme created and used for each session is available with all the details at https://doi.org/10.5281/zenodo.16490124 (accessed on 27 July 2025).

The CG followed the standard physical education curriculum as prescribed by the Italian National Guidelines [76,77]. This programme, designed to promote general physical and psychological well-being, included bodyweight exercises, group activities using small equipment, and joint mobility exercises.

### 2.5. Statistical Analysis

All statistical analyses were conducted using IBM SPSS Statistics software (Version 29). Initially, independent samples *t*-tests were performed to compare baseline scores between the EG and the CG for each outcome measure, in order to assess any pre-existing differences. Prior to these analyses, the distributions of the outcome variables were examined through descriptive statistics, skewness and kurtosis indices. Most variables demonstrated values within acceptable limits (±2), indicating approximate normality. For instance, the Locomotor Score (skewness = −0.15 CG, 0.14 EG) and Ball Skills Score (skewness = −0.12 CG, −0.01 EG) both showed near-symmetric distributions, while the Corsi Forward Task and Corsi Backward Task showed mild deviations (skewness = 1.09 and 1.39 in CG, respectively). Given the overall sample size (*n* = 184) and the relatively balanced groups, these deviations were not considered to compromise the validity of subsequent parametric analyses. In line with previous methodological evidence [78], ANCOVA and related F-tests are generally robust to moderate violations of normality when sample sizes are adequate. To further ensure validity, we also checked the homogeneity of variances (Levene’s test) and the homogeneity of regression slopes (group × covariate interactions), and the study design guaranteed independence of observations.

## 3. Results

No significant differences were observed between the experimental and control groups in demographic characteristics at baseline. Additionally, the groups were comparable across all cognitive and motor outcome measures at baseline.

Table 2 presents the mean scores and standard deviation for both groups at baseline and post-intervention, allowing for a comparison of performance across the assessed domains.

All participants attended 95% of the scheduled coordination-based physical activity sessions and successfully completed all components of the intervention protocol.

The analysis of visuospatial working memory showed significant differences between the EG and the CG. In the *Corsi Forward* task, the EG obtained a significantly higher mean score (*M* = 5.35, *SD* = 0.55) compared to the CG (*M* = 4.53, *SD* = 0.85), (*F*(1, 178) = 115.47, *p* < 0.001) with a very large effect size (*d* = 1.12). Gender showed a statistically significant effect (*F* = 7.98, *p* = 0.005), though its impact was less pronounced than that of the intervention. Among the covariates included (gender, BMI, and weekly physical activity), gender emerged as a significant factor in the VSWM tasks. Specifically, girls slightly outperformed boys on both the *Corsi Forward* (F(1, 178) = 7.98, *p* = 0.005) and *Corsi Backward* tasks (F(1, 178) = 6.36, *p* = 0.013). In contrast, BMI and weekly physical activity were not significantly associated with either of the visuospatial memory outcomes.

**Figure 2:**
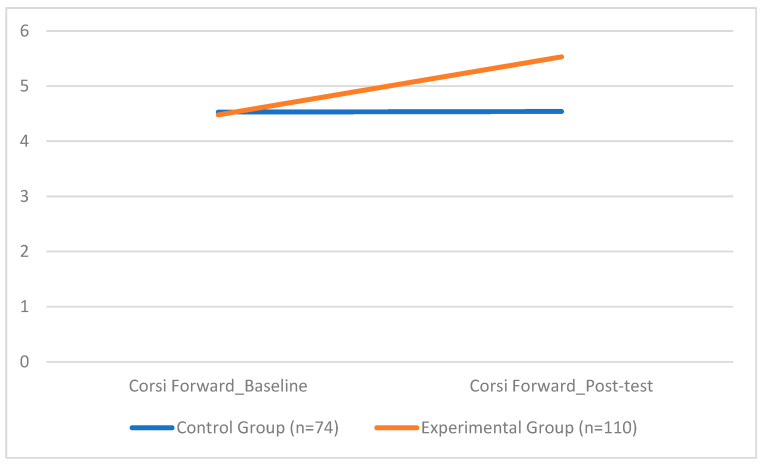
Group Differences in Corsi Forward Test Scores.

Figure 2 illustrates the statistically significant difference between the experimental and control groups on the *Corsi Forward* task, highlighting the cognitive benefit of the coordination training protocol.

In the *Corsi Backward* test, the EG also demonstrated significantly higher performance compared to the CG, with a higher mean score (*M* = 5.31, *SD* = 0.46 vs. M = 4.09, *SD* = 0.81) (*F*(1, 178) = 250.96, *p* < 0.001). The intervention effect was substantial, as reflected in a very large effect size (*d*= 1.40). Gender had a statistically significant, though less pronounced, effect on performance (*F* = 6.36, *p* = 0.013). None of the covariates, BMI, gender, or total weekly training hours, showed significant associations with visuospatial working memory outcomes (all *p* > 0.05).

**Figure 3:**
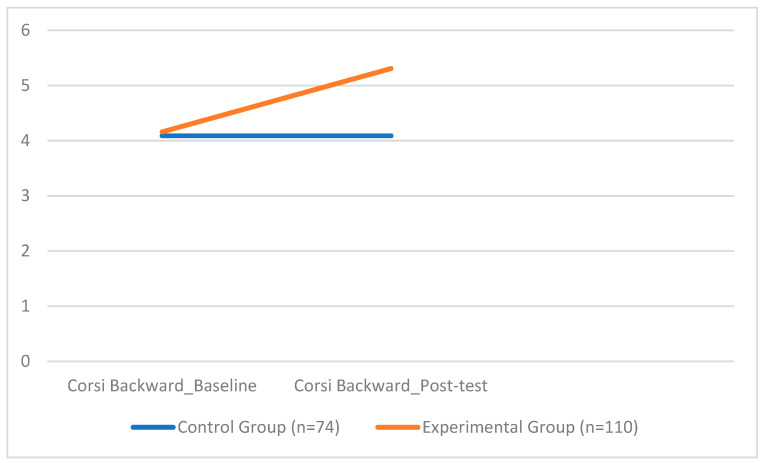
Group Differences in Corsi Backward Test Scores.

Figure 3 illustrates the statistically significant difference between the experimental and control groups in the *Corsi Backward* task, further supporting the efficacy of the coordination-based intervention on cognitive performance.

**Figure 4:**
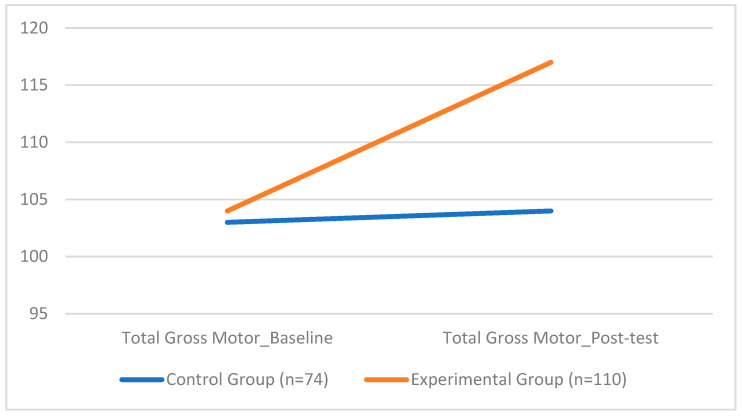
Group Differences in Total Gross Motor.

For the Total Gross Motor, the ANCOVA revealed a significant group effect (*F*(1, 177) = 386.73, *p* < 0.001), with the EG displaying significantly higher scores (*M* = 116.67, *SD* = 3.82) compared to the CG (*M* = 103.51, *SD* = 5.73) with a large effect size (*d*=1.58) (see Figure 4). Gender did not show a significant influence on motor performance (*F* = 2.06, *p* = 0.153). None of the covariates, including BMI, gender, and total weekly training hours, were found to significantly affect gross motor outcomes (all *p* > 0.05).

Specifically, for the score of the locomotor subscale, the ANCOVA revealed a significant group effect (*F*(1, 177) = 628.54, *p* < 0.001), with a huge effect size (*d* = 2.11). The EG achieved significantly higher scores (*M* = 46.86, *SD* = 1.52) compared to the CG (*M* = 38.97, *SD* = 3.08).

**Figure 5:**
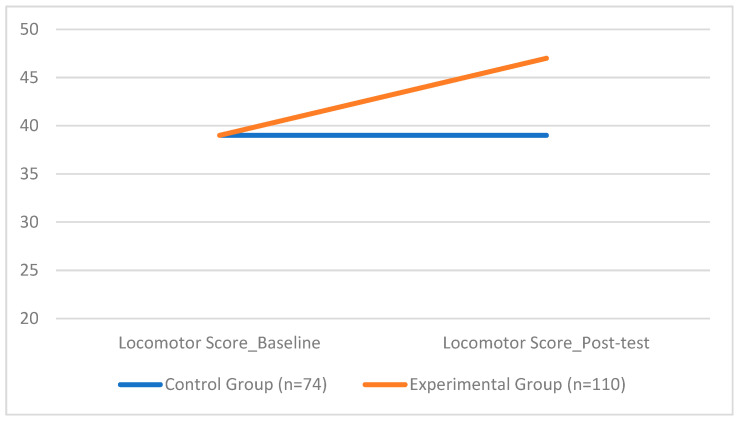
Group Differences in Locomotor Scores.

Figure 5 illustrates the difference between the experimental and control groups on the locomotor subscale score, further emphasising the positive impact of the coordination-based intervention on fundamental movement skills.

For ball skills score, the EG demonstrated significantly higher adjusted performance (*M* = 48.79, *SD* = 1.25) compared to the CG (*M* = 42.74, *SD* = 2.42). The ANCOVA revealed a robust main effect of the group, F(1, 177) = 556.07, *p* < 0.001, with a very large effect size (*d* = 1.34). The model also showed a significant effect of the baseline score, F(1, 177) = 47.86, *p* < 0.001, and a modest but significant effect of gender, F(1, 177) = 4.64, *p* = 0.033, with girls performing slightly better. In contrast, BMI and weekly activity were not significant predictors.

**Figure 6:**
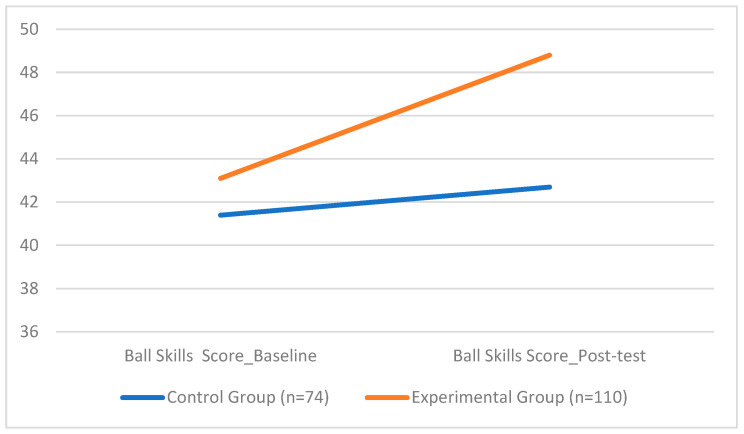
Group Differences in Ball Skills Scores.

Figure 6 displays the difference between the experimental and control groups in the ball skills score, underscoring the intervention’s effectiveness in enhancing object control and coordination.

## 4. Discussion

This study aimed to evaluate the effectiveness of a structured coordination-based physical activity intervention in promoting the development of cognitive and motor skills in primary school children. Specifically, the research sought to determine whether participation in a well-defined motor programme could enhance visuospatial working memory and gross motor competence when compared to a control condition following the standard physical education curriculum. The results demonstrated that the coordination-focused intervention had a significant and positive effect on both cognitive performance—particularly visuospatial working memory—and motor proficiency, aligning with evidence from existing literature [51,79,80,81,82].

Across all outcome measures, children in the experimental group outperformed their peers in the control group, supporting the efficacy of coordination-based activities in stimulating broader developmental processes. Crucially, the study found that Body Mass Index (BMI) and total weekly training hours outside the intervention were not significant covariates in the final analyses. Although the literature suggests that healthy-weight children may benefit more cognitively due to higher fitness levels and more frequent engagement in physical activity [83,84,85,86,87], our findings indicate that the coordination training produced benefits independent of BMI status in this sample.

With regard to cognitive outcomes, significant improvements were observed in both the Corsi Forward and Corsi Backward tasks in the experimental group. The *Forward* test, assessing short-term spatial memory, showed an increase in mean score from 4.48 to 5.35 in the experimental group, while the control group’s performance remained largely unchanged. These results suggest that participation in coordination-focused physical activity enhances children’s ability to encode and retain basic spatial sequences. The *Backward* test, which requires mental manipulation of spatial information and thus places greater demands on executive functioning, yielded even more pronounced results. The experimental group improved from a mean of 4.16 to 5.31, indicating a substantial enhancement in core executive processes such as working memory, inhibition, and cognitive flexibility. These findings support a growing body of research that highlights the cognitive benefits of engaging in physically and cognitively demanding motor activities [82,88,89,90,91,92,93,94]. More specifically, improvements observed in the *Forward* condition reflect gains in attention and spatial encoding [95,96], while the enhanced performance in the *Backward* condition aligns with studies demonstrating that complex motor tasks, those requiring high coordination and dynamic balance, can stimulate executive functions [97,98]. Given that the intervention was explicitly designed to challenge children’s neuromotor systems through varied and progressive coordinative tasks, it is plausible that these cognitive benefits arose from the integration of motor planning, sequencing, and sensorimotor adaptation inherent in the activities. The working memory improvements observed in this study align with previous research on the cognitive benefits of coordination training. Ref. [99] Arslan-Kabasalak et al. (2023) found that postural control, a core aspect of coordination, was closely linked to dynamic balance in youth, reinforcing the connection between motor and cognitive performance. This suggests that interventions like ours, targeting coordination, can produce meaningful cognitive effects during development.

Further support comes from a recent meta-analysis [100], which showed that cognitively demanding physical activities, including coordinative tasks, significantly improve working memory in children (SMD = 0.34). Interventions longer than six weeks with structured sessions, like ours, were particularly effective. These findings highlight how coordination exercises, involving simultaneous motor and spatial demands, may enhance key working memory functions such as updating and active information maintenance.

A significant sex effect was also found in both versions of the visuospatial memory task in both *forward* and *backward* tasks, though it was smaller than the effect of the intervention itself. This finding partially aligns with existing literature on gender differences in visuospatial processing. While some studies report that girls may show advantages in early cognitive tasks [100,101], other research indicates that boys typically outperform girls in a variety of visuospatial tasks throughout childhood [102,103,104,105]. These mixed results suggest that gender differences may be influenced by a combination of developmental, cultural, and contextual factors, warranting further exploration.

Analysis of motor performance revealed equally significant improvements following the coordination-based intervention. The Total Gross Motor increased substantially in the experimental group, from 104 to 117, while remaining essentially stable in the control group (103 to 104). This group difference was supported by a large effect size, highlighting the effectiveness of the intervention in enhancing gross motor skills. Detailed analyses of the motor subcomponents reinforced this trend. Both locomotor and manipulation skills showed statistically significant improvements in the experimental group, with particularly high F-values (*F* = 628.54 for locomotion; *F* = 556.07 for manipulation, all *p* < 0.001). These results confirm that coordination training contributes meaningfully to the development of gross motor competence in school-aged children. They align with existing research showing that structured physical activity supports the maturation of fundamental movement skills [69,106,107,108]. Importantly, performance on the Total Gross Motor was consistent across genders, supporting prior findings that suggest minimal or no gender differences in gross motor performance at this developmental stage, particularly around age 9 when such differences tend to converge [109]. Similarly, the observed improvements in locomotor and manipulation domains reflect the capacity of structured physical activity to stimulate both dynamic body coordination and hand–eye coordination [110,111,112].

The study’s internal validity is strengthened by the high adherence rate to the intervention (95% of sessions completed) and the absence of significant baseline differences between groups. Although sex was statistically significant for some individual measures, its influence was consistently smaller than the effect of the intervention itself, confirming that the observed gains were primarily attributable to the structured coordination programme. These findings further support emerging evidence that physical exercise—particularly when cognitively demanding and coordinative—can exert broad benefits across both motor and cognitive domains. Such benefits are likely mediated by neuroplasticity and enhanced executive functioning. Notably, children appear to benefit most from structured movement experiences that integrate cognitive demands, offering a holistic developmental stimulus [113,114,115,116,117,118].

The motor improvements seen in the EG align with training specificity principles, as the intervention included fundamental skills also assessed by the TGMD-3. However, these gross motor skills (e.g., running, jumping, throwing) are also part of standard physical education and likely present in the CG activities. This suggests that the observed gains were not due to test-specific practice, but to the added cognitive and coordinative demands of the intervention. Importantly, the cognitive improvements in the EG, despite no direct training on memory tasks, point to a potential transfer effect, supporting the broader value of motor-cognitive programmes in educational settings.

The present study specifically investigated the effects of a coordination-based physical activity programme on gross motor skills and visuo-spatial working memory in primary school children. The use of validated and standardised tools, the TGMD-3 and the BVS-Corsi-2 (Italian version), ensured both the reliability of the measurements and their feasibility in applied educational settings. Post-intervention results demonstrated significant improvements in both motor and cognitive domains, extending the literature supporting the efficacy of coordination-focused programmes for enhancing executive functions. In particular, the intervention—designed to engage children in spatiotemporal challenges and complex motor control—appears to have facilitated gains in the visuospatial components of working memory. These findings resonate with recent neuroscientific models that underscore the embodied nature of cognition, challenging the traditional mind–body dualism. Rather than operating in isolation, cognitive processes are increasingly understood to be shaped by dynamic bodily interaction with the environment [119]. This embodied connection remains relevant into adulthood, where maintaining and strengthening working memory is essential for coping with complex tasks, managing everyday life, and supporting lifelong learning [120].

However, this study presents several limitations. First, the sample, although adequate for preliminary analyses, was drawn from a single school context, which may limit the generalisability of the findings. Second, the relatively short duration of the intervention (16 weeks) may not have been sufficient to fully capture potential long-term or sustained effects. Third, the study focused exclusively on one component of executive function, visuospatial working memory, leaving open the question of whether coordination-based interventions might also influence other key executive processes, such as inhibitory control or cognitive flexibility. Fourth, although both groups were taught by instructors with equivalent qualifications in Sports Science, differences in teaching style and the novelty of the experimental programme may have influenced participant engagement and cannot be fully controlled. Other limitations include individual variability in motor and cognitive development and the exclusion of psychosocial factors (e.g., motivation, self-efficacy, enjoyment, self-esteem) that may influence participation and outcomes. Future studies should also examine the relationship between VSWM and different sports (e.g., gymnastics, swimming, team sports) to assess whether specific activities differentially affect motor and cognitive development. Despite these limitations, the findings hold substantial relevance for educational practice. The intervention, designed to be easily implemented within existing school frameworks, illustrates how structured coordination-based physical activities can serve as an effective means of promoting both motor and cognitive development. These results support the view that physical education, when approached from an integrated and developmentally informed perspective, has the potential to foster not only physical well-being but also cognitive competencies critical for academic learning, beginning as early as the preschool years [121,122]. Future research should aim to build upon these findings by involving larger and more diverse samples, extending the intervention period, and incorporating broader assessments of executive function and related cognitive domains. Moreover, exploring the potential transfer of motor-cognitive improvements to academic performance, particularly in subjects such as mathematics or science, where visuo-spatial abilities are foundational, would offer valuable insights into the broader educational impact of such programmes.

## 5. Conclusions

In conclusion, this study provides preliminary yet promising evidence of the effectiveness of coordination-based physical activity interventions in enhancing specific cognitive functions, particularly visuo-spatial working memory, among primary school children. These findings contribute to a growing body of research advocating for the integration of physical and cognitive development within the school curriculum and offer a compelling foundation for rethinking the role of motor activity in educational settings.

## Figures and Tables

**Table 1:** Demographic Characteristics of Participants by Study Group.

	Control Group (CG)		Experimental Group (EG)	
**Girl**	43	41.9%	51	46.4%
**Boy**	31	58.1%	59	53.6%
	M (SD)		M (SD)	
**Age**	9.50 (0.50)		9.47 (0.55)	
**Body Mass Index**	18.7 (3.89)		19.3 (3.37)	
**Total weekly hours of physical activity**	3.07 (1.65)		3.50 (1.50)	

**Table 2:** Analysis of Covariance.

	Experimental			Control			Main Effect Group	
Dependent Variable	Baseline, M (SD)	Post-Test, M (SD)	Percentage of Change	Baseline, M (SD)	Post-Test, M (SD)	Percentage of Change	Analysis of Covariance	Effect Size
Corsi Forward Task	4.48 (0.70)	5.35 (0.55)	19.4	4.53 (0.85)	4.54 (0.80)	0.2	*F* = 115.47, *p* < 0.001	1.12
Corsi Backward Task	4.16 (0.76)	5.31 (0.46)	27.6	4.09 (0.90)	4.09 (0.73)	0.0	*F* = 250.96, *p* < 0.001	1.40
Total Gross Motor	104 (8.04)	117 (3.82)	12.5	103 (6.85)	104 (5.73)	0.9	*F* = 386.73, *p* < 0.001	1.58
Locomotor Score	39 (3.88)	46.9 (1.52)	20.2	38.9 (3.38)	39 (3.08)	0.2	*F* = 628.54, *p* < 0.001	2.11
Ball Skills Score	43.1 (2.95)	48.8 (1.25)	13.2	41.4 (3.71)	42.7 (2.42)	3.1	*F* = 556.07, *p* < 0.001	1.34

*Note. Effect size = Cohen’s d (pooled standard deviation).* Degrees of freedom may vary slightly across outcomes due to missing data on specific measures.

## Data Availability

The data supporting the findings reported in this study are available at the link https://doi.org/10.5281/zenodo.17198144 (accessed on 25 September 2025).

## Abbreviations

The following abbreviations are used in this manuscript:

EFs	Executive Functions
PA	Physical Activity
PE	Physical Education
VSWM	Visualspatial Working Memory
TGMD	Test of Gross Motor Development
BMI	Body Mass Index
PDPAR	Previous Day Physical Activity Recall
EG	Experimental Group
CG	Control Group
